# Evaluation of Individual Cardiovascular Risk in Pre-Dialysis CKD Patients by Using the Ratio of Calcium–Phosphorus Product to Estimated Glomerular Filtration Rate (Ca × P/eGFR)

**DOI:** 10.3390/biomedicines13010235

**Published:** 2025-01-19

**Authors:** Krasimir Kostov, Tatyana Simeonova, Borislav Ignatov, Tsvetelina Eftimova

**Affiliations:** 1 Department of Physiology and Pathophysiology, Medical University-Pleven, 1 Kliment Ohridski Str., 5800 Pleven, Bulgaria; dr_tsimeonova@abv.bg (T.S.); dr_bignatov@abv.bg (B.I.); 2 Hemodialysis Unit, St. Marina Hospital, 5800 Pleven, Bulgaria; dr_eftimova@abv.bg

**Keywords:** chronic kidney disease, cardiorenal syndrome type 4, cardiovascular risk, biomarkers of kidney function

## Abstract

Background: Chronic kidney disease (CKD) patients have an increased risk of cardiovascular disease (CVD), necessitating effective risk assessment methods. This study evaluates the calcium–phosphorus product (Ca × P) to estimated glomerular filtration rate (Ca × P/eGFR) ratio as a potential biomarker for predicting CV risk in pre-dialysis CKD patients. Methods: Eighty-four CKD patients in stages G1–G4, according to the KDIGO criteria, were classified into CVD (*n* = 43) and non-CVD (*n* = 41) groups. Biochemical parameters, including serum creatinine (SCr), blood urea nitrogen (BUN), calcium (Ca), inorganic phosphate (Pi), parathyroid hormone (PTH), alkaline phosphatase (ALP), Ca × P, eGFR, and the Ca × P/eGFR ratio, were measured and calculated. Statistical analyses were performed to identify predictors of CV risk and evaluate the diagnostic reliability of the Ca × P/eGFR ratio for predicting the risk. Results: Significant differences were observed in SCr, BUN, eGFR (*p* < 0.001), and the Ca × P/eGFR ratio (*p* = 0.007) between the groups. Regression analysis indicated the Ca × P/eGFR ratio as a significant CVD risk predictor (*p* = 0.012, OR = 1.206, 95% CI: 1.042–1.395). Receiver Operating Characteristic (ROC) curve analysis revealed an AUC of 0.751 (*p* < 0.001, 95% CI: 0.645–0.857), with a sensitivity and specificity of the method of 74.4% and 70.7%, respectively. Significant correlations were found between the Ca × P/eGFR ratio and SCr, BUN, UA, Ca, Pi, PTH, and ALP. Conclusions: The Ca × P/eGFR ratio may serve as a significant predictor of CVD risk in pre-dialysis CKD patients, suggesting that its integration into routine evaluations could enhance CV risk stratification and management.

## 1. Introduction

Chronic kidney disease (CKD) is a progressive condition affecting over 10% of the global population [1]. Patients with CKD have an increased risk of cardiovascular (CV) events and mortality even in the early stages of the disease (stages G1–G3), which significantly increases in the advanced stages (stages G4 and G5), where about 50% of deaths are related to CV diseases (CVD) [2]. CKD results in a chronic pro-inflammatory state that contributes to vascular and myocardial remodeling, accelerating the aging of the CV system, with hypertension further enhancing this process [3,4]. This is a prerequisite for the development of atherosclerosis, vascular calcification, myocardial fibrosis, and heart valve calcification, which are major factors for CV events and mortality in patients with CKD [5,6,7,8]. Often, the engagement of the CV system in the course of CKD is termed chronic renocardiac syndrome, or cardiorenal syndrome type 4 (CRS type 4) [9,10,11,12]. The management of CRS type 4 should include not only control of renal function but also active assessment and management of CVD risk to reduce mortality and improve the quality of life of patients [13]. In this article, we propose an innovative approach to assess CVD risk in pre-dialysis CKD patients by using the ratio of calcium–phosphorus product (Ca × P) to estimated glomerular filtration rate (Ca × P/eGFR) [14,15]. This method enables the calculation of CV risk using routine indicators, making the assessment more accessible and easier to apply in daily clinical practice.

## 2. Materials and Methods

### 2.1. Study Design and Participants

This study involved 84 patients (32 men and 52 women) in pre-dialysis CKD stages (G1–G4), according to the Kidney Disease: Improving Global Outcomes (KDIGO) criteria [16]. The stage of the disease was determined based on the estimated glomerular filtration rate (eGFR), which was calculated using the Modification of Diet in Renal Disease (MDRD) equation [17]. The study population was divided into two groups: a group with CVD (CVD group, *n* = 43) and a group without CVD (non-CVD group, *n* = 41). The data on CVD, including hypertension (HTN), coronary artery disease (CAD), heart failure (HF), cardiac valve calcification (CVC), peripheral arterial disease (PAD), and arrhythmias, were confirmed through a comprehensive cardiological examination that included blood pressure measurement, electrocardiograms, echocardiography, angiography, and a Doppler ultrasound. Patients with acute kidney injury, end-stage kidney disease (stage G5), liver disease, and active inflammatory diseases or infections, and those who had undergone kidney transplantation, were excluded from this study. This research was conducted in accordance with the ethical principles for medical research as specified in the Declaration of Helsinki and received approval from the Research Ethics Committee of Medical University-Pleven.

### 2.2. Biochemical Analysis

Blood samples were collected in the morning after overnight fasting and were centrifuged at 2500 rpm for 10 min to separate the serum. The concentrations of serum creatinine (SCr), blood urea nitrogen (BUN), uric acid (UA), calcium (Ca), inorganic phosphate (Pi), parathyroid hormone (PTH), and alkaline phosphatase (ALP) were measured using Roche Cobas E 311 and Cobas E 411 analyzers.

### 2.3. Calculation of Ca × P/eGFR Ratio


(1) $$Ca\times P/eGFRRatio=\frac{Ca\times P}{eGFR}=\frac{({mmol/L)}^{2}}{\left(mL/min/1.73m^{2}\right)}$$

(2) $$Ca\times P/eGFRRatio(Percentage)=\frac{Ca\times P}{eGFR}\times 100=\frac{({mmol/L)}^{2}\times 100}{\left(mL/min/1.73m^{2}\right)}$$

Given the small numerical values of the Ca × P/eGFR ratio, expressing it as a percentage enhances the interpretability of the results and provides a better presentation of the data.

### 2.4. Statistical Analysis

Statistical analyses were conducted using SPSS version 23.0 (SPSS, Inc., Chicago, IL, USA). Data were assessed for normality of distribution using the Shapiro–Wilk test. One-way ANOVA was performed to evaluate differences between the means of the groups. Binary logistic regression analysis was employed to identify individual predictors of CVD risk. After testing for multicollinearity, the statistically significant variables were introduced into the multivariate binary logistic regression to identify the independent risk factors using the forward stepwise selection method. The results are presented as the odds ratio (OR), 95% confidence interval (CI), and *p*-value. The Receiver Operating Characteristic (ROC) curve analysis was used to evaluate the diagnostic performance of the Ca × P/eGFR ratio in predicting the risk of CVD. The results are expressed as the area under the curve (AUC), *p*-value, and 95% CI. The optimal cut-off value was determined using Youden’s index [18]. Pearson’s correlation coefficient was used to determine significant relationships between the Ca × P/eGFR ratio and the other baseline variables. Statistical significance was considered at *p* < 0.05.

## 3. Results

### 3.1. Baseline Patient Characteristics

Significant differences were observed in the eGFR (*p* < 0.001), SCr (*p* < 0.001), BUN (*p* < 0.001), and the Ca × P/eGFR ratio (*p =* 0.007) between groups with and without CVD. Other variables, including UA, Ca, Pi, Ca × P, PTH, and ALP, did not show significant differences between the two groups (Table 1).

### 3.2. Ca × P/eGFR Ratio as a Predictor of CVD Risk

**Figure 1:**
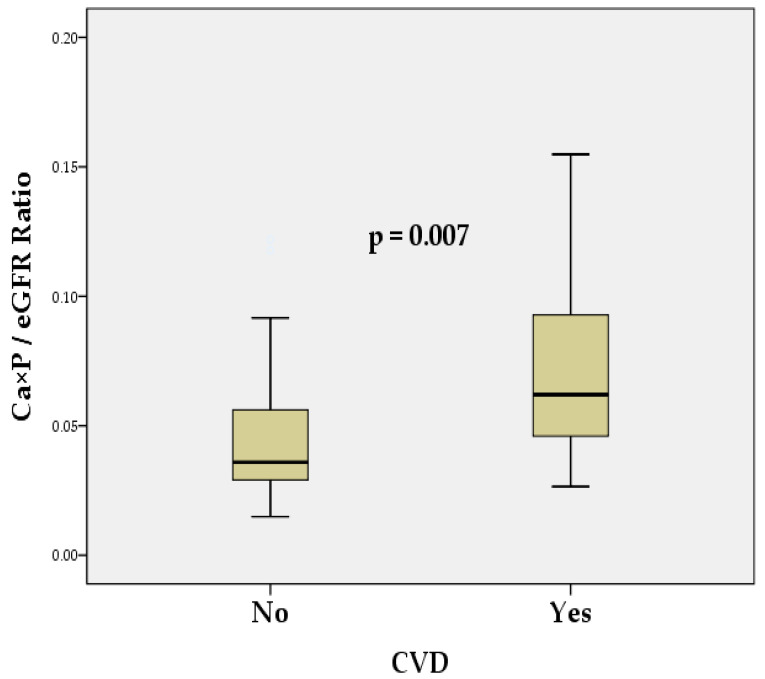
Comparison of the Ca × P/eGFR ratio between patients with and without CVD in the entire CKD cohort. Ca × P/eGFR is presented in (mmol/L)^2^/(mL/min/1.73 m^2^). No—non-CVD group, Yes—CVD group, *p* < 0.05, statistically significant.

The Ca × P/eGFR ratio was statistically significantly higher in patients with CVD compared to those without CVD (*p* = 0.007). The non-CVD group (*n* = 41) had a mean ratio of 0.05 ± 0.04 (mmol/L)^2^/(mL/min/1.73 m^2^), while the CVD group (*n* = 43) had a mean ratio of 0.07 ± 0.04 (mmol/L)^2^/(mL/min/1.73 m^2^). This suggests that the Ca × P/eGFR ratio is elevated in patients with CVD, indicating a potential association between this ratio and increased CV risk (Figure 1).

The binary logistic regression analysis identified several predictors of CVD risk in pre-dialysis CKD patients. In the univariate analysis, elevated levels of BUN (*p* = 0.002) and SCr (*p* = 0.001) were significant predictors of CVD risk, with higher levels associated with an increased risk (OR = 1.317, 95% CI: 1.109–1.563 for BUN; OR = 1.018, 95% CI: 1.007–1.029 for SCr). Lower eGFR levels were significantly associated with a higher risk of CVD (*p* < 0.001, OR = 0.963, 95% CI: 0.944–0.982). Additionally, the Ca × P/eGFR ratio was also identified as a significant predictor of CVD risk (*p* = 0.012). The odds ratio (OR) for the Ca × P/eGFR ratio was 1.206 (95% CI: 1.042–1.395), indicating that higher values of the Ca × P/eGFR ratio are associated with an increased risk of CVD. Specifically, for each unit increase in the Ca × P/eGFR ratio, the odds of developing CVD increase by approximately 20.6%. This finding suggests that the Ca × P/eGFR ratio could be a valuable marker for assessing CVD risk in CKD patients, as it reflects the combined impact of calcium–phosphorus levels and renal function on CV health. In the multivariate analysis, only the eGFR remained a significant predictor of CVD risk (*p* < 0.001, OR = 0.963, 95% CI: 0.944–0.982). This indicates that lower eGFR levels are independently associated with a higher risk of CVD in the CKD cohort. The Ca × P/eGFR ratio, although not an independent predictor in the multivariate analysis, showed a significant association with CVD risk in the univariate analysis, highlighting its potential as a marker for CV risk assessment in CKD patients. These findings underscore the importance of monitoring eGFR levels and considering the Ca × P/eGFR ratio in the management and risk assessment of CVD in CKD patients (Table 2).

**Figure 2:**
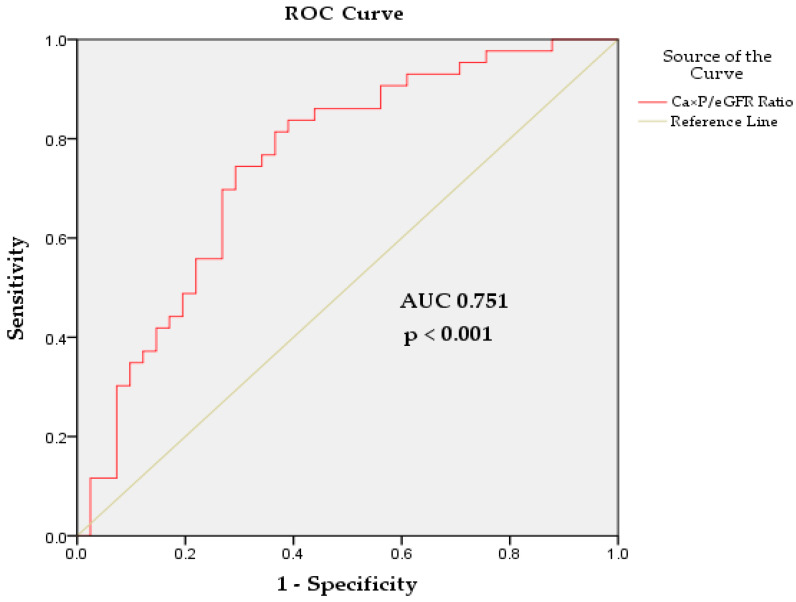
ROC curve plot illustrating the diagnostic capability of the Ca × P/eGFR ratio to evaluate CV risk in CKD patients before dialysis. Abbreviations: ROC, Receiver Operating Characteristic; AUC, area under the curve; *p*, probability value.

To evaluate the diagnostic performance of the Ca × P/eGFR ratio in predicting the risk of CVD, ROC analysis was used. The ROC curve demonstrated a significant AUC of 0.751 (*p* < 0.001, 95% CI: 0.645–0.857), indicating a relatively good discriminatory ability to distinguish between patients at a high and low risk of CVD. A cut-off value of 0.048 (mmol/L)^2^/(mL/min/1.73 m^2^) was identified as optimal, with a sensitivity of 74.4% and a specificity of 70.7%. This means that at this cut-off, the test correctly identifies 74.4% of patients who will develop CVD (true positives) and correctly identifies 70.7% of patients who will not develop CVD (true negatives). Overall, these results support the use of the Ca × P/eGFR ratio as a valuable tool for CVD risk stratification in CKD patients before dialysis (Figure 2).

The Pearson correlation analysis revealed significant correlations between the Ca × P/eGFR ratio and various indicators of renal function. The strongest positive correlation was observed between the Ca × P/eGFR ratio and SCr (r *=* 0.841, R^2^ *=* 0.707, *p* < 0.001). A strong negative correlation was found between the Ca × P/eGFR ratio and the eGFR (r *=* –0.796, R^2^ *=* 0.633, *p* < 0.001). Significant positive correlations were also observed between the Ca × P/eGFR ratio and BUN (r *=* 0.723, R^2^ *=* 0.523, *p* < 0.001), Ca (r *=* 0.491, R^2^ *=* 0.241, *p* < 0.001), Pi (r *=* 0.458, R^2^ *=* 0.210, *p* < 0.001), and ALP (r *=* 0.399, R^2^ *=* 0.159, *p* < 0.001). Moderate positive correlations were observed between the Ca × P/eGFR ratio and UA (r *=* 0.264, R^2^ *=* 0.070, *p =* 0.043) and PTH (r *=* 0.366, R^2^ *=* 0.134, *p =* 0.016). The marked correlations between the Ca × P/eGFR ratio and various parameters of renal function highlight the potential of the Ca × P/eGFR ratio as an important indicator for evaluating renal function and its influence on CV health in CKD patients (Table 3).

## 4. Discussion

The development of CRS type 4 is most often attributed to the following four pathophysiological mechanisms: (1) disturbances in the calcium-and-phosphorus metabolism, resulting in medial vascular calcification and calcification of the heart valves; (2) pro-inflammatory and pro-fibrotic changes associated with CKD, leading to conditions such as atherosclerosis, coronary artery disease, myocardial fibrosis, heart failure, and arrhythmias; (3) complications of renal hypertension affecting the CV system, including myocardial infarction, stroke, and heart failure; and (4) other accompanying disorders, such as anemia, volume overload, and electrolyte imbalance, leading to increased cardiac workload and arrhythmogenesis.

The identification of appropriate biomarkers that reflect these mechanisms poses a significant challenge. The biomarkers can be any measurable parameter, such as components of serum or urine, or other variables, which may facilitate diagnosis and monitoring of the progression of chronic renocardiac syndrome [11]. The development of CRS type 4 leads to neurohormonal activation (mainly of the renin–angiotensin–aldosterone system), hemodynamic changes, chronic inflammation, oxidative stress, and endothelial dysfunction, which ultimately result in damage and fibrosis of the heart and vessels [9,19,20,21,22,23]. In this regard, biomarkers can reflect, either alone or in combination, each one of these pathological processes. The biomarkers that have been found to be associated with CRS type 4 to date include brain natriuretic peptide (BNP), N-terminal pro-brain natriuretic peptide (NT-proBNP), cardiac troponins, urotensin II, galectin-3, creatinine, microalbuminuria, uric acid, cystatin C, homocysteine, and aldosterone [22,24,25].

When assessing CV risk in patients with CKD, it is crucial to consider not only the traditional set of biomarkers but also new approaches that integrate various aspects of the interplay between renal and CV pathology. Commonly used biomarkers for evaluating CV risk in CKD patients include SCr [26,27,28,29,30,31,32], eGFR [33,34,35,36,37,38,39], proteinuria [40,41,42,43,44], lipid profiles [45,46,47], homocysteine [48,49,50], C-reactive protein (CRP) [51,52,53], and others, which provide essential data regarding renal function and inflammation. SCr and the eGFR play a fundamental role in the assessment of renal function, but neither of these biomarkers offers direct insight into how imbalances in mineral metabolism in CKD affect the CV health of patients. Proteinuria is another important marker indicating renal impairment and is associated with increased CV morbidity, yet it also lacks a direct connection to mineral metabolism. Lipid profiles, encompassing levels of LDL and HDL, are important for coronary heart disease assessment but do not comprehensively reflect the complex array of risk factors associated with CKD. Homocysteine, which is linked to metabolic disorders and nutrition, demonstrates associations with an elevated CV risk but is not a specific indicator of renal function. CRP serves as a marker of inflammation and may act as a precursor for CVD and events; however, it similarly fails to provide clear insights into renal function or mineral metabolism. In the context of these traditional markers, the Ca × P/eGFR ratio offers a more integrated approach, allowing for the monitoring of renal function while also assessing how imbalances in mineral metabolism impact CV health. This integrative measure underscores the importance of a holistic evaluation of patients with CKD to better understand and manage their CV risk.

The Ca × P/eGFR ratio we suggest represents a marker that unifies various aspects of the pathophysiological processes associated with CRS type 4. It integrates information about the calcium–phosphorus metabolism with information about renal function, which is expressed through the eGFR. The ratio helps assess mineral metabolism in the body, which is particularly important in patients with CKD, where an imbalance of calcium and phosphate can lead to bone and CV complications [54,55,56,57,58,59]. This imbalance can cause the calcification of soft tissues, including arteries and heart valves, which significantly increases the risk of CVD [6,7,60,61,62,63,64,65,66]. High levels of calcium–phosphate product (Ca × P) are also associated with a greater likelihood of developing vascular calcification and an increased CV risk [67,68,69,70,71,72,73]. On the other hand, a progressive decline in the eGFR is associated with an increased likelihood of CV events, such as myocardial infarction, stroke, and heart failure [33,34,35,36,37,38,39]. Patients with a reduced eGFR often have secondary hypertension, which is an additional risk factor for CVD [74,75,76].

The results of our study showed significant differences in the eGFR (*p* < 0.001), SCr (*p* < 0.001), and BUN (*p* < 0.001) between groups with and without CVD. However, no significant differences were found in other variables such as Ca, Pi, Ca × P, UA, PTH, and ALP between the two groups (Table 1). Multivariate regression analysis revealed that reduced renal function (a lower eGFR) is the main independent risk factor for CVD in patients with CKD (*p* < 0.001, OR = 0.963) (Table 2). Data for the Ca × P/eGFR ratio showed that it was significantly increased in patients with CVD compared to those without CVD (*p* = 0.007) (Figure 1). The mean ratio in the non-CVD group was 0.05 ± 0.04 (mmol/L)^2^/(mL/min/1.73 m^2^), whereas in the CVD group it was 0.07 ± 0.04 (mmol/L)^2^/(mL/min/1.73 m^2^). Univariate regression analysis identified the Ca × P/eGFR ratio as a significant predictor of CVD risk (*p* = 0.012), with an OR of 1.206, highlighting its potential as a marker for CV risk (Table 2). ROC analysis demonstrated that the Ca × P/eGFR ratio has a relatively good discriminatory ability to distinguish between patients at a high and low risk of CVD (AUC 0.751, *p* < 0.001), with a sensitivity of 74.4% and a specificity of 70.7% (Figure 2). Significant correlations were also found between the Ca × P/eGFR ratio and various indicators of renal function, revealing a close association between the Ca × P/eGFR ratio and renal performance (Table 3).

Based on the results obtained for the Ca × P/eGFR ratio, we developed a scale to measure CV risk in pre-dialysis CKD patients. This scale includes six risk categories ranging from “very low risk” to “extremely high risk” (Table 4).

The scale may serve multiple clinical purposes. It allows for the early identification of patients at an increased risk of CV complications, leading to more effective risk management and prevention of serious events. Physicians can implement personalized treatment and prevention strategies, including dietary changes, medication, and monitoring. The scale can also be used to monitor disease progression over time, aiding in the assessment of the current treatment’s effectiveness and the need for therapeutic adjustments. Additionally, informing patients about their risk classification can increase their awareness and motivate them to follow recommendations for risk reduction.

Notably, our research is the first to utilize the Ca × P/eGFR ratio as a marker for CV risk. Implementing this ratio in clinical practice may optimize CVD management for CKD patients, allowing physicians to identify high-risk individuals for more effective monitoring and targeted interventions.

Despite the significant findings, our study has several limitations. The sample size is relatively small, which limits the generalizability of the conclusions. Additionally, the study design is cross-sectional, which does not allow for causal inferences to be drawn. Future studies with a larger sample size and a prospective design may confirm our findings and provide a deeper understanding of the relationship between the Ca × P/eGFR ratio and CVD risk in patients with CKD.

## 5. Conclusions

In summary, our study demonstrates that the Ca × P/eGFR ratio is a reliable predictor of CV risk in pre-dialysis CKD patients. By integrating data on calcium–phosphorus metabolism and renal function, this ratio offers a holistic strategy for CV risk assessment. The findings underscore that the monitoring of the Ca × P/eGFR ratio in clinical practice could substantially improve the early identification and management of high-risk patients. Additionally, this study establishes a foundation for future research aimed at refining and validating this predictive tool in various clinical settings.

## Figures and Tables

**Table 1:** Baseline characteristics of patients in the study groups.

Variables	All Patients (*n* = 84)	Non-CVD Group (*n* = 41)	CVD Group (*n* = 43)	*p*-Value (ANOVA)
Age, years ^1^	64.71 ± 12.95	64.70 ± 13.82	64.72 ± 12.22	0.996
Male/Female, *n*/*n*	32/52	11/30	21/22	–
SCr, µmol/L ^1^	114.65 ± 52.92	93.73 ± 52.03	134.60 ± 46.06	<0.001
BUN, mmol/L ^1^	8.27 ± 3.78	6.79 ± 2.79	9.69 ± 4.08	<0.001
UA, µmol/L ^1^	313.22 ± 97.23	283.43 ± 87.15	332.25 ± 99.68	0.059
eGFR, mL/min/1.73 m^2 1^	61.60 ± 27.79	74.09 ± 27.42	49.69 ± 22.63	<0.001
CKD 1, *n* (%) ^2^	15 (18)	12 (29)	3 (7)	–
CKD 2, *n* (%) ^2^	25 (30)	16 (39)	9 (21)	–
CKD 3, *n* (%) ^2^	32 (38)	10 (25)	22 (51)	–
CKD 4, *n* (%) ^2^	12 (14)	3 (7)	9 (21)	–
HTN, *n* (%)	43 (51)	–	43 (100)	–
CAD, *n* (%)	19 (23)	–	19 (44)	–
HF, *n* (%)	13 (15)	–	13 (30)	–
CVC, *n* (%)	18 (21)	–	18 (42)	–
PAD, *n* (%)	8 (10)	–	8 (19)	–
Arrhythmias, *n* (%)	4 (5)	–	4 (9)	–
Ca, mmol/L ^1^	2.49 ± 0.21	2.51 ± 0.29	2.48 ± 0.10	0.444
Pi, mmol/L ^1^	1.16 ± 0.17	1.13 ± 0.15	1.18 ± 0.18	0.202
Ca × P, mmol^2^/L^2^ ^1^	2.90 ± 0.51	2.86 ± 0.53	2.94 ± 0.50	0.480
PTH, mmol/L ^1^	84.39 ± 50.39	89.07 ± 67.41	82.13 ± 41.02	0.677
ALP, mmol/L ^1^	72.44 ± 19.54	70.53 ± 18.98	74.25 ± 20.11	0.387
Ca × P/eGFR Ratio, (mmol/L)^2^/(mL/min/1.73 m^2^) ^1^	0.06 ± 0.04	0.05 ± 0.04	0.07 ± 0.04	0.007

^1^ Mean ± SD; ^2^ CKD stages. Abbreviations: SCr, serum creatinine; BUN, blood urea nitrogen; UA, uric acid; eGFR, estimated glomerular filtration rate; HTN, hypertension; CAD, coronary artery disease; HF, heart failure; CVC, cardiac valve calcification; PAD, peripheral arterial disease; Ca, calcium; Pi, inorganic phosphate; Ca × P, calcium–phosphorus product; PTH, parathyroid hormone; ALP, alkaline phosphatase. *p* < 0.05, statistically significant.

**Table 2:** Binary logistic regression analysis identifying predictors of CVD risk in the general CKD cohort, based on the presence-versus-absence of CVD.

Variables	Univariate Analysis * (Single Predictors)			Multivariate Analysis ** (Predictors in Model)		
	Coefficient (B)	OR (95% CI)	*p*-Value	Coefficient (B)	OR (95% CI)	*p*-Value
BUN, mmol/L	0.275	1.317 (1.109–1.563)	0.002	–	–	–
SCr, µmol/L	0.018	1.018 (1.007–1.029)	0.001	–	–	–
eGFR, mL/min/1.73 m^2^	–0.038	0.963 (0.944–0.982)	<0.001	–0.038	0.963 (0.944–0.982)	<0.001
Ca × P/eGFR Ratio, (mmol/L)^2^/(mL/min/1.73 m^2^)	0.187	1.206 (1.042–1.395)	0.012	–	–	–

* Method = Enter, ** Method = forward stepwise (likelihood ratio). Abbreviations: OR, odds ratio; CI, confidence interval; BUN, blood urea nitrogen; SCr, serum creatinine; eGFR, estimated glomerular filtration rate; Ca × P, calcium–phosphorus product. *p* < 0.05, statistically significant.

**Table 3:** Correlations between the Ca × P/GFR ratio and various indicators of renal function.

Correlations	Pearson Correlation Coefficient (r)	R-Squared (R^2^)	*p*-Value
Ca × P/eGFR and SCr	0.841 **	0.707	<0.001
Ca × P/eGFR and eGFR	–0.796 **	0.633	<0.001
Ca × P/eGFR and BUN	0.723 **	0.523	<0.001
Ca × P/eGFR and UA	0.264 *	0.070	0.043
Ca × P/eGFR and Ca	0.491 **	0.241	<0.001
Ca × P/eGFR and Pi	0.458 **	0.210	<0.001
Ca × P/eGFR and PTH	0.366 *	0.134	0.016
Ca × P/eGFR and ALP	0.399 **	0.159	<0.001

* Correlation is significant at the 0.05 level, ** Correlation is significant at the 0.01 level. Abbreviations: Ca × P/eGFR, calcium–phosphorus-product-to-estimated-glomerular-filtration-rate ratio; SCr, serum creatinine; BUN, blood urea nitrogen; UA, uric acid; Ca, calcium; Pi, inorganic phosphate; PTH, parathyroid hormone; ALP, alkaline phosphatase.

**Table 4:** Ca × P/eGFR ratio scale for evaluation of CVD risk in pre-dialysis CKD patients.

Risk Classification	Ca × P/eGFR Ratio *	Ca × P/eGFR Ratio (%) *	Risk Description
Very Low Risk	<0.03	<3	Very low likelihood of CV complications
Low Risk	0.03–0.5	3–5	Low likelihood of CV complications
Moderate Risk	0.05–0.10	5–10	Moderate likelihood of CV complications
High Risk	0.10–0.15	10–15	Increased likelihood of CV complications
Very High Risk	0.15–0.20	15–20	Significant likelihood of serious CV events
Extremely High Risk	>0.20	>20	Critical likelihood of severe CV events

* Ca × P/eGFR ratio and Ca × P/eGFR ratio (percentage) are presented in (mmol/L)^2^/(mL/min/1.73 m^2^) and (mmol/L)^2^ × 100/(mL/min/1.73 m^2^), respectively.

## Data Availability

The authors confirm that the data supporting the findings of this report are available within the article.
